# Complement evasion factor (CEF), a novel immune evasion factor of *Streptococcus pyogenes*

**DOI:** 10.1080/21505594.2022.2027629

**Published:** 2022-01-30

**Authors:** Haniyeh Aghababa, Yi Tian Ting, Devaki Pilapitiya, Jacelyn M.S. Loh, Paul G. Young, Thomas Proft

**Affiliations:** a Department of Molecular Medicine & Pathology, School of Medical Sciences, The University of Auckland, Auckland, New Zealand; b School of Biological Sciences, the University of Auckland, Auckland, New Zealand; c Infection and Immunity Program, Biomedicine Discovery Institute, Monash University, Clayton, Australia; d University of Melbourne, The Peter Doherty Institute for Infection and Immunity, Melbourne, Australia; e Maurice Wilkins Centre for Biomolecular Discoveries. The University of Auckland, Auckland, New Zealand

**Keywords:** Streptococcus pyogenes, Group A Streptococcus, immune evasion, complement, complement deposition, *Galleria mellonella* infection model, glycan-binding

## Abstract

*Streptococcus pyogenes*, a leading human pathogen, is responsible for a wide range of diseases, including skin and soft tissue infections and severe invasive diseases. *S. pyogenes* produces a large arsenal of virulence factors, including several immune evasion factors. We have identified an open reading frame (*spy0136*) in the *S. pyogenes* SF370 genome encoding a protein of unknown function. Using recombinant Spy0136 in a pull-down assay with human plasma and ELISA, we have identified four complement proteins (C1r, C1s, C3, and C5) as binding partners. Treatment of the complement proteins with PNGase F abrogated binding to C1s, C3, and C5, indicating glycan-dependent interactions. rSpy0136 inhibited complement-mediated hemolysis and interfered with all three complement pathways in a Wieslab complement assay. Furthermore, rSpy0136 inhibited deposition of the C3b opsonin and the membrane attack complex (MAC) on the surface of *S. pyogenes*. We therefore named the previously unknown protein ‘complement evasion factor’ (CEF).

An *S. pyogenes Δspy0136/cef* deletion mutant showed decreased virulence in an *in-vitro* whole blood killing assay and a *Galleria mellonella* (wax moth) infection model. Furthermore, an *L. lactis spy0136/cef* gain-of-function mutant showed increased survival during growth in whole human blood. Analysis of serum samples from patients with invasive *S. pyogenes* revealed Spy0136/CEF sero-conversion indicating expression during disease. In summary, we have identified a novel *S. pyogenes* immune evasion factor that binds to several complement proteins to interfere with complement function. This is the first example of a *S. pyogenes* virulence factor binding to several different target proteins via glycan-dependent interactions.

## Introduction

*Streptococcus pyogenes* (Group A Streptococcus) is a Gram-positive coccus that exclusively infects humans and causes a variety of diseases ranging from pharyngitis, tonsillitis, and skin infections to severe invasive diseases, such as streptococcal toxic shock syndrome [1–3]. Infections can also result in post-streptococcal autoimmune sequelae like acute rheumatic fever, rheumatic heart disease, and acute glomerulonephritis [4–6]. It is estimated that *S. pyogenes* causes more than 1.7 Mio infections each year with approximately 500,000 deaths [7].

One reason for *S. pyogenes* being a highly successful pathogen is a large arsenal of secreted and surface-anchored virulence factors such as adhesins, pili, cytolysins, spreading factors and immune evasion factors [8–12]. *S. pyogenes* secretes a variety of highly mitogenic exotoxins (superantigens) that stimulate large numbers of T cells and antigen presenting cells. This results in massive release of pro-inflammatory cytokines sometimes referred to as a ‘cytokine storm’ and can lead to systemic inflammation and multiorgan failure [13,14]. *S. pyogenes* also developed several strategies to evade the host immune response. This includes secreted and cell wall-anchored DNases to destroy neutrophil extracellular traps (NETs) [15–17], streptolysin O (SLO) and streptolysin S (SLS) to lyse leukocytes [18], and cytokine-targeting enzymes such as the cysteine protease SpeB (cleaving interleukin (IL)-1β) [19] and the serine protease SpyCEP (cleaving IL-8) [20,21].

The complement system is important in host defense against *S. pyogenes*. There are three independent pathways that converge to form the complement protein C3b which binds to (opsonises) the bacteria [22]. Additional complement proteins are also added to form the terminal C5b-9 complex or membrane attack complex (MAC). This also results in the release of C5a, a chemotactic factor that attracts neutrophils to the site of infection [23]. The three complement pathways are initiated by a) binding of IgG or IgM antibodies to the bacterial cell surface triggering the formation of the C1 complex consisting of C1q, C1r and C1s (classical pathway), b) binding of the mannose-binding lectin (MBL) to mannose residues on the bacterial surface (lectin pathway) or c) the spontaneous low-level hydrolysis of C3 to generate C3b in fluid phase (alternative pathway) [24,25].

Several *S. pyogenes* immune evasion factors target the complement system, highlighting its importance.^10^ *S. pyogenes* prevents the initiation of the classical pathway by secreting immunoglobulin-degrading enzymes (IdeS/Mac-1, Mac-2 and EndoS) [26–28] or the C1q inhibitor endopeptidase O (PepO) which binds C1q with a higher affinity than IgG thereby preventing initiation of the classical pathway by blocking IgG binding to C1q [29]. Streptococcal inhibitor of complement (SIC) binds strongly to C5b67 and C5b678 complexes and to a lesser extent C5b-9 preventing insertion of the MAC into the membrane, although the functional role of MAC in the destruction of Gram-positive bacteria remains unclear [30]. *S. pyogenes* also secretes C5a peptidase (ScpA) [31] to inactivate the chemotactic C5a preventing neutrophil recruitment. Recently, it was shown that ScpA also cleaves C3 and C3a [32]. The multifunctional virulence factor SpeB is also able to degrade opsonising IgG and several complement proteins, including C2, C3 and C4 [33]. Several cell surface-expressed proteins, like M protein, fibronectin-binding protein FbaA and streptococcal collagen-like protein 1 (Scl1) recruit the complement regulator factor H (FH) reducing C3b deposition on the surface of *S. pyogenes* [34,35].

We have identified a novel *S. pyogenes* gene product (Spy0136) and show that Spy0136 interferes with complement formation by specifically binding to several complement proteins. Hence, we named this novel protein “complement evasion factor” (CEF).

## Materials and methods

### Bacterial strains, media, and antibiotics

*Streptococcus pyogenes* SF370 (M1/T1 serotype) was grown in Brain Heart Infusion (BHI) broth at 37°C under static conditions. *Lactococcus lactis* MG1363 was cultured in GM17 medium supplemented with 0.5% glucose at 28°C, and *E. coli* BL21(DE3)pLysS (Novagen) was grown in Luria-Bertani (LB) broth at 37°C with agitation. The cultures were supplemented with appropriate antibiotics at the following concentrations: ampicillin (50 µg/ml for *E. coli*), spectinomycin (100 µg/ml for *S. pyogenes* and *E.coli*), chloramphenicol (30 μg/ml for *E. coli* BL21), and kanamycin (50 µg/ml for *E. coli*; 200 µg/ml for *L. lactis* and *S. pyogenes*).

### Cloning and recombinant protein production

The *cef* gene lacking the region encoding for the predicted signal peptide sequence was amplified from genomic DNA isolated from *S. pyogenes* SF370 by PCR using the primers CEF-proEXHta.fw/rev (Table 1) and iProof polymerase (Bio-Rad, Hercules, CA, USA). The DNA fragment was cloned into the KasI and XhoI restriction sites of a pProExHta plasmid (Invitrogen) that has been modified to encode Maltose binding protein (MBP) as an affinity/solubilization tag (His_6_-MBP-rTEV-CEF) [36]. For recombinant protein expression, *E. coli* BL21 (DE3) cells were transformed with CEF plasmid and grown with Luria-Bertani (LB) broth supplemented with 100 μg/ml ampicillin at 37°C until the optical density at 600 nm (OD_600_) reached 0.6. The cultures were induced with 0.1 mM isopropyl-β-D-1-thiogalactopyranoside (IPTG) at 18°C for 16 h, and the cells were harvested by centrifugation. Cell pellets were resuspended in a lysis buffer (50 mM Tris-Cl [pH 8.0], 300 mM NaCl, 10 mM imidazole), lysed by sonication and insoluble matter sedimented by centrifugation. The fusion protein was purified from the soluble phase by immobilized-metal affinity chromatography (IMAC) on a nickel-charged nitrilotriacetic acid resin (Ni^2+^-NTA sepharose, Bio-Rad) according to the manufacturer’s instruction. The His_6_-MBP affinity tag was cleaved from the CEF recombinant protein with recombinant tobacco etch virus protease (rTEV-His_6_, 1:25 ratio) and concurrently dialyzed to remove Imidazole. Recombinant CEF was separated from the rTEV-His_6_ protease and uncleaved protein by subtractive IMAC. Further purification of the CEF protein was achieved by size-exclusion chromatography using a Superdex 200 Increase 10/300 GL column (GE Healthcare, Chicago, U.S.) equilibrated on PBS. The purified protein was estimated to be approximately 99% pure as indicated by SDS-PAGE analysis.

**Table 1.** Oligo primers used for PCR amplification (restriction enzyme cleavage sites are shown in bold)

Primer	Sequence 5’ – 3’	Restriction site
*spy0136*-proExHta.fw	ggc**ggcgcc**aaacatgataacattgagaaag	KasI
*spy0136*-proExHta.rev	aaa**ctcgag**ttactcaccgaattcccc	XhoI
*spy0136*-FR1.fw	gcacgc**gtcgac**ctgattctgtcagaggaacac	SalI
*spy0136*-FR1.rev	gc**tctaga**gctagtagtgaagtgaagag	XbaI
*spy0136*-FR2.fw	aa**ctgcag**ctgccagtccatcatcttttg	PstI
*spy0136*-FR2.rev	ccc**cccggg**ctaacagtatactggaggattg	XmaI
*spy0136*-seq.fw	gcttcactcatcaacaatatctttg	-
*spy0136*-seq.rev	gcaatcatgttgtcttagactc	-
*aad9*.fw	ccttattggtacttacatgtttg	-
*aad9*.rev	ccattcaatattctctccaag	-
*spy0136*-comp.fw	ctag**ctcgag**ttggaggaataaaaaatg aaacgatgtaataaatatctc	XhoI
*spy0136*-comp.rev	gc**ggatcc**aattactcaccgaattccccttta	BamHI

### Coupling of protein to sepharose

Recombinant CEF at a concentration of 2 mg/ml in PBS (pH 8.0) was mixed with Cyanogen bromide (CNBr)-activated sepharose 4B (GE Healthcare, Chicago, U.S.) overnight at 4°C. The remaining active sites on the beads were then blocked in 1 ml 100 mM Tris, pH 8.0 at RT for 2 h. After washing the beads three times with PBS (pH 8.0), the protein-coupled sepharose beads were stored in PBS (pH 8.0) with 0.025% NaN_3_ at 4°C.

### Pull-down assay and mass spectrometry (LC-MS/MS) analysis

One hundred microliters of the freshly prepared human plasma was diluted to 500 μl using TSA buffer (10 mM Tris-HCl pH 8.0, 140 mM NaCl, 0.025% (v/w), NaN_3_) and mixed with 10 μl protein-coupled sepharose by rotating at 4°C for 30 min. The unbound proteins were removed by centrifugation at 16,000 g for 1 min at 4°C. Beads were washed three times with RIPA buffer (1% (w/v) sodium deoxycholate, 0.1% (w/v) SDS, 500 mM NaCl, 1% (v/v) Triton X-100, 10 mM Tris HCl, pH 8.0). For the final wash step, the sepharose beads were washed in TSA, and the excess liquid was removed using a Hamilton syringe. The protein-bead complex was resuspended in 10 µl SDS-PAGE reducing buffer and analyzed on a 12.5% SDS-PAGE. Coomassie blue-stained protein bands of interest were excised from the gel, diced, destained, and dehydrated with acetonitrile (Ajax Finechem, Auckland, NZ), then reduced with 5 mM dithiothreitol (Bio-Rad, Carlsbad, CA), alkylated with 15 mM iodoacetamide (Sigma-Aldrich, St Louis, MO) in the dark for 30 minutes at room temperature, and digested with 12.5 ng/ul modified sequencing grade porcine trypsin (Promega, Madison, WI) at 45°C for 1 hour. The digest was acidified with formic acid and then injected onto a 0.3 × 10 mm trap column and desalted for 3 minutes at 30ul/min before being separated on a 0.3 × 100 mm 3.5um Zorbax 300SB C18 Stablebond column (Agilent Technologies, Santa Clara, CA, USA) using the following gradient at 6 μl/min: 0–3 min 10%B, 24 min 50%B, 26 min 97%B, 29 min 97%B, 30.5 min 10%B, 35 min 10%B, where A was 0.1% formic acid in water and B was 0.1% formic acid in acetonitrile. The column eluate was ionized in the electrospray source of a QSTAR-XL Quadrupole Time-of-Flight mass spectrometer (Applied Biosystems, Foster City, CA, USA). A TOF-MS scan from 330–1600 m/z was performed, followed by three rounds of MS/MS on the most intense multiply-charged precursors in each cycle. The resulting MS/MS data was searched against a protein sequence database the human entries from Uniprot.org (89,796 entries at the time), using ProteinPilot version 5.0 (AB SCIEX).

Human plasma was collected from consented donors and approved by the University of Auckland Human Participants Ethics Committee (Reference number 021200).

### ELISA with complement proteins

A Nunc MaxiSorpTM Immuno-assay plate (Sigma-Aldrich) was coated with 1 µg/well of rCEF in 100 µl ELISA coating buffer (15 mM Na_2_CO_3_, 35 mM NaHCO_3_, 0.01% NaN_3_, pH 9.6) overnight at 4°C. The same quantity of human serum albumin (HSA) was applied to the control wells. Following three washes with PBS-T (0.05% Tween-20 in PBS), the wells were blocked with 200 µl of 3% BSA in PBS at 37°C for 2 h. After the wash steps, plasma proteins C1s, C1r, C3, C5 (all purified from human plasma, Merck, Kenilworth, U.S.), C2 (recombinant protein expressed in HEK293, Abnova, Taipei, Taiwan), or C4BPA (recombinant protein expressed from a wheat germ *in-vitro* system, Abnova) at different concentrations (0, 0.25, 0.5, 1.0, 1.5, and 2 μg/well) in ELISA dilution buffer (50 mM NaCl, 20 mM Tris–HCl, pH 7.4, 1 mM CaCl_2_) were added to each well and incubated for 2 h at 37°C. The plate was then incubated with the appropriate primary antibody (1:1,000, Abcam, Cambridge, U.K.) and HRP-conjugated anti-rabbit IgG antibody (1:10,000, Abcam) at RT for 3 h and 1 h, respectively. Subsequent to the final wash, ELISAs were developed with 100 μl TMB substrate (Pierce, Appleton, U.S.), and the reaction was stopped by adding 100 μl of 1 M HCl. The absorbance was measured at 450 nm using an EnSightTM Multimode plate reader (Perkin Elmer, Waltham, U.S.). To remove N-linked glycans, complement proteins were treated with PNGase F according to the manufacturer’s instruction (New England Biolabs, Ipswich, U.S.). Three independent experiments on duplicates were carried out.

### Complement-mediated hemolytic assay

Fresh sheep blood (5 ml) was centrifuged at 1,250 g for 5 min at 4°C, and the erythrocytes were resuspended in Alsever's solution (Sigma-Aldrich, St. Louis, US) at a 1:1 ratio. The 5 ml solution containing red blood cells (RBCs) was mixed with 40 ml ice-cold 1x Gelatine HEPES buffer GHB^++^ (GHB^++^ 5x:1 M NaCl, 400 mM HEPES, 0.66% (w/v) Bovine Skin Type B Gelatin, pH: 7.35 plus 2 mM MgCl_2_, and 0.3 mM CaCl_2_) and incubated at 37°C for 15 min to lyse unstable cells. Following lysis, erythrocytes were washed in ice-cold 0.15 M NaCl until the supernatant turned clear and resuspended in 1x GHB^++^ to form a 2% (v/v) suspension. RBCs were sensitized by combining equal volumes of 2% RBCs and anti-sheep red blood cell stroma antibody (Abcam) at a ratio of 1 to 2,500 in 1x GHB^++^ for 30 min at 37°C. Hemolytic assays were performed in Nunc 96-well round-bottom plates (Sigma-Aldrich) in a total volume of 250 μl. Guinea pig serum (Vernon Jansen Unit, The University of Auckland, 100 µl) as the source of complement was diluted to the desired concentration using 1x GHB^++^ and pre-incubated with 50 µl of CEF for 30 min at 37°C. Following this step, 100 µl sensitized RBCs were added to the mixture and incubated for 1 h at 37°C with occasional agitation to keep RBCs in suspension. The unlysed erythrocytes were pelleted by centrifugation at 1,250 g for 5 min at 4°C. The supernatant was transferred to a flat-bottom 96-well plate, and hemolysis was measured in triplicates on an EnSight^TM^ Multimode plate reader (Perkin Elmer) at 412 nm.

### Complement pathway ELISA

The Wieslab® total complement screen kit is a commercial ELISA-based test system for the functional assessment of the three individual pathways of the complement system. The test was performed according to the manufacturer’s instructions. First, serum was pre-incubated with rCEF at different concentrations (0, 0.125, 0.25, 0.5, 1, 2 µM) for 30 min at 37°C. The C5b-9 complex (membrane attack complex, MAC) was then detected with a specific alkaline phosphatase labeled antibody to the neoantigen of the MAC complex using an EnSight^TM^ Multimode plate reader (Perkin Elmer) at 405 nm. Complement activity was calculated using the following formula:

*% Complement activity = 100% × Mean A_405_ (sample) – Mean A_405_ (inactivated serum)/Mean A_405_ (standard serum) – Mean A_405_ (inactivated serum).*

Two independent tests were carried out in duplicates.

### Complement deposition assay

Complement deposition was analyzed by flow cytometry. *S. pyogenes* was grown to mid-exponential phase (OD_600nm_ ~ 0.5). After a wash with PBS, 300 µl bacterial suspensions (5x10^6^ CFU) in HEPES buffered saline (HBS^++^: 20 mM HEPES, 140 mM NaCl, pH 7.4, 2.5 mM MgCl_2_, 5 mM CaCl_2_) containing 0.01% BSA were mixed with 300 µl freshly prepared human serum containing rCEF for 50 min at 37°C with constant shaking. The mixture was then washed twice with 1.5 ml PBS supplemented with 1% BSA. The pellet was resuspended in 300 µl of PBS-1% BSA containing 5 µg/ml of either rabbit anti-C5b-9 or rabbit anti-C3b antibodies (Abcam) and incubated at RT for 15 min. Subsequent to the wash step, the bacteria were resuspended in 300 µl of FITC-labeled secondary antibody (Abcam) at a ratio of 1:100 in PBS-1% BSA and incubated for 15 min at RT. Finally, the bacteria were washed and resuspended in 800 µl of PBS for flow cytometry analysis using a BD LSRII flow cytometer (BD Biosciences). An opsonised sample containing bacteria and serum and a non-opsonised sample (bacteria only) were used as controls.

### Generation of knockout and complemented strains

A *S. pyogenes cef* gene-knockout mutant was generated by allelic replacement with a spectinomycin resistance gene (*aad9)* using the pFW11 plasmid [37] (a gift from Andreas Podbielski, University of Rostock). DNA sequences representing the upstream flanking region (FR1) and downstream flanking region (FR2) of the *cef* gene (~1,000 bp each) were amplified from genomic *S. pyogenes* SF370 DNA by PCR using iProof™ high-fidelity DNA polymerase (Bio-Rad, Hercules, CA, USA) and primers Spy0136-FR1.fw/rev and Spy0136-FR2.fw/rev (Table 1) and cloned into the pFW11 MCS-I site and MCS-II site, respectively. The recombinant construct was electroporated into *S. pyogenes* SF370 using a Bio-Rad Gene Pulser, and the transformants were selected on BHI agar plates containing spectinomycin. Replacement of the *cef* gene with *aad9* was subsequently confirmed by PCR using Spy0136-seq.fw/rev and aad9.fw/rev primers (Table 1). In addition, validation of the deletion mutants was performed at the protein level by Western blotting.

The *S. pyogenes Δcef* deletion mutant was complemented with the complete *cef* gene cloned into the pLZ12-Km2 plasmid [38] (a gift from Nobuhiko Okada, Kitasato University, Japan) using the primers Spy0136-comp.fw/rev (Table 1).

To generate an *L. lactis* gain-of-function mutant, pLZ12-Km2:*cef* was introduced into *L. lactis* MG1363 by electroporation. Expression of CEF in *S. pyogenes* and *L. lactis* strains was confirmed by Western blotting using rabbit anti-CEF antiserum.

### Whole blood killing assay

Different strains of *S. pyogenes* (Table 1) were grown in BHI to late exponential phase (OD_600nm_ ~ 0.8). Approximately 10^5^ CFU of bacteria in a volume of 50 µl were incubated with 1 ml of fresh heparinized human whole blood from a nonimmune consented donor for 3 h at 37°C with constant rotation. Every 30 min, 100 μl samples were taken, serially diluted and plated onto BHI agar plates in triplicates to enumerate the surviving bacteria. The percentage of survival was calculated as *[CFU (at a given time point)/CFU (at the start)] x100*.

*L. lactis* grown to the late exponential phase was collected and resuspended in Hank’s Balanced Salt Solution (HBSS). Approximately 10^3^ bacteria were added to 1 ml of blood and incubated for 3 h at 37°C. Serially diluted samples were plated on GM17 agar plates in triplicates for enumeration. Three independent experiments were carried out.

### Galleria mellonella infection model

A group of 10 antibiotic-free, healthy (with no markings) and medium-sized (~1.5 cm) *Galleria mellonella* larvae (Biosuppliers-New Zealand) were chosen for each experiment. 1.5 × 10^6^ CFU of different *S. pyogenes* strains in a volume of 20 µl were injected into the lower-left proleg of wax moth larvae using an insulin syringe (BD Biosciences, USA). The injected dose was confirmed by culturing the samples on plates for colony enumeration. Each group of larvae was incubated at 37°C in a Falcon®12-well plate (Biocampare, San Francisco, U.S.) without food and monitored daily for the following features up to five days post-infection: activity, cocoon formation, melanization, and survival. A health index score [39] between 0 to 10 based on these attributes was determined for each larva.

### Western blot analysis

Protein samples were run on an SDS-PAGE gel alongside a pre-stained ladder (Invitrogen, Waltham, U.S.) and then transferred onto a nitrocellulose membrane (Bio Rad Laboratories, Hercules, U.S.) in transfer buffer (25 mM Tris-HCl, 192 mM glycine, 20% (v/v) methanol, pH 8.3) using a TE77 semi-dry transfer unit (Hoefer, Holliston, U.S.) at 200 V, 50 mA/gel for 1 h. The membrane was incubated by blocking solution (TBS-T plus 5% (w/v) skim milk powder) on a shaker at RT for at least 1 h to block nonspecific binding sites of the membrane. Following this step, the membrane was washed twice with TBS-T (20 mM Tris-Cl, 150 mM NaCl, pH 7.6, 0.1% (v/v) Tween-20) for 5 min. The membrane was probed with human sera from patients with invasive GAS infections and healthy donors at 1/100 dilution in probing solution (TBS-T plus 2.5% (w/v) skim milk powder) for 1 h at RT. The membrane was then washed three times with TBS-T for 5 min each and incubated with HRP-conjugated goat anti-human IgG (Abcam) in a probing buffer and incubated for 1 h followed by washing with TBS-T three times for 5 min. Western blots were developed with ECL detection reagent (Amersham Biosciences) and analyzed using a ChemiDoc^TM^ imaging system (BioRad).

### Human sera

Serum samples were collected at Middlemore Hospital, Auckland, New Zealand, between July 2001 and January 2003 with approval by the Auckland Human Ethics Committee.

### Statistical analysis

Statistical analysis was carried out using GraphPad Prism software version V7.03. Statistical significance was calculated using t-tests to compare two data sets and one-way ANOVA with Tukey’s multiple comparisons test. A P-value of <0.05 was considered significant. The Kaplan–Meier estimator in GraphPad Prism was used to estimate the survival function.

## Results

### Bioinformatic analysis of Spy0136/CEF

We have recently investigated the *S. pyogenes* pilus structure [40,41], which is encoded by several genes (*spy0125* to *spy0130*) within the highly variable fibronectin-binding, collagen-binding T antigen (FCT) region [42]. As virulence genes are often clustered on bacterial genomes, we have analyzed the genomic region downstream of the FCT region in the SF370 strain (serotype M1) and identified a gene (*spy0136*) encoding for a hypothetical protein of unknown function (NCBI accession number AAK33244). The protein consists of 221 amino acids with a predicted N-terminal signal peptide (SP) sequence (27 aa) and no other hydrophobic region suggesting that the protein is secreted into the environment. BLAST searches revealed that the *spy0136/cef* gene is highly conserved in *S. pyogenes* and was found in >1,000 *S. pyogenes* genomes encoding proteins with aa sequence identities between 85.5% and 100%. Interestingly, a protein of unknown function and 70.1–92% percent sequence identity to Spy0136/CEF was found in several strains of the dog pathogen *Streptococcus canis*. No significant sequence similarities were found with proteins of any other species.

### Identification of CEF-binding proteins

**Figure 1.**
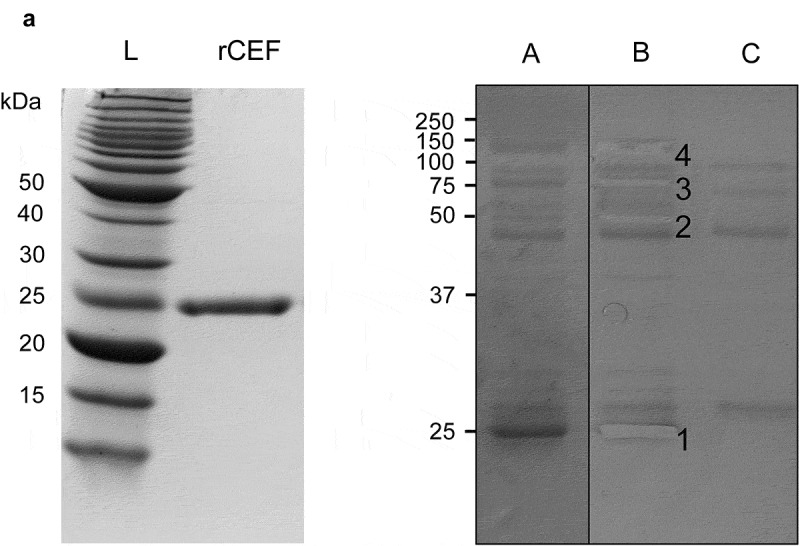
**Identification of plasma proteins binding to rSpy0136/CEF**. A. Recombinant CEF was expressed in *E. coli* and purified by immobilized metal affinity chromatography (IMAC), and size exclusion chromatography. The purity of rCEF was confirmed by SDS-PAGE. B. Purified rCEF was immobilized on sepharose beads and used for a pull-down experiment with human plasma. (a) Washed beads were loaded on a SDS-PAGE gel. (b) Gel slices extracted for mass spectrophotometry analysis. Mass-spectrometry results identified proteins: (1) rCEF, (2) fibrinogen β-chain, (3) C1r complement protein, C1s complement protein, Fibulin-1, (4) C4BPA, C3 complement protein. (c) Negative control of uncoupled Sepharose beads.

The *cef* gene without the region encoding the predicted SP sequence was expressed in *E. coli* as a soluble fusion protein with maltose-binding protein (MBP). The fusion protein was purified by Ni^2+^-NTA affinity chromatography, and MBP was removed using rTEV protease. CEF was further purified by size-exclusion chromatography and yielded 0.95 mg of recombinant protein per liter of *E. coli* culture with a high level of purity (Figure 1a).

In an attempt to identify potential binding partners, rCEF was immobilized on sepharose beads and used in a pull-down experiment with human plasma. Several of the plasma proteins identified by mass spectrometry are involved in the complement pathway. The four complement factors are C1r, C1s, C3, and C4BPA, with C1r/C1s and C4BPA providing the highest peptide coverage and intensity of signal (Figure 1b, Supplementary file). The plasma proteins fibulin-1 and fibrinogen β-chain also gave high peptide coverage and signal intensity. Keratin was dismissed as a contaminant frequently identified in pull-down experiments with plasma.

**Figure 2.**
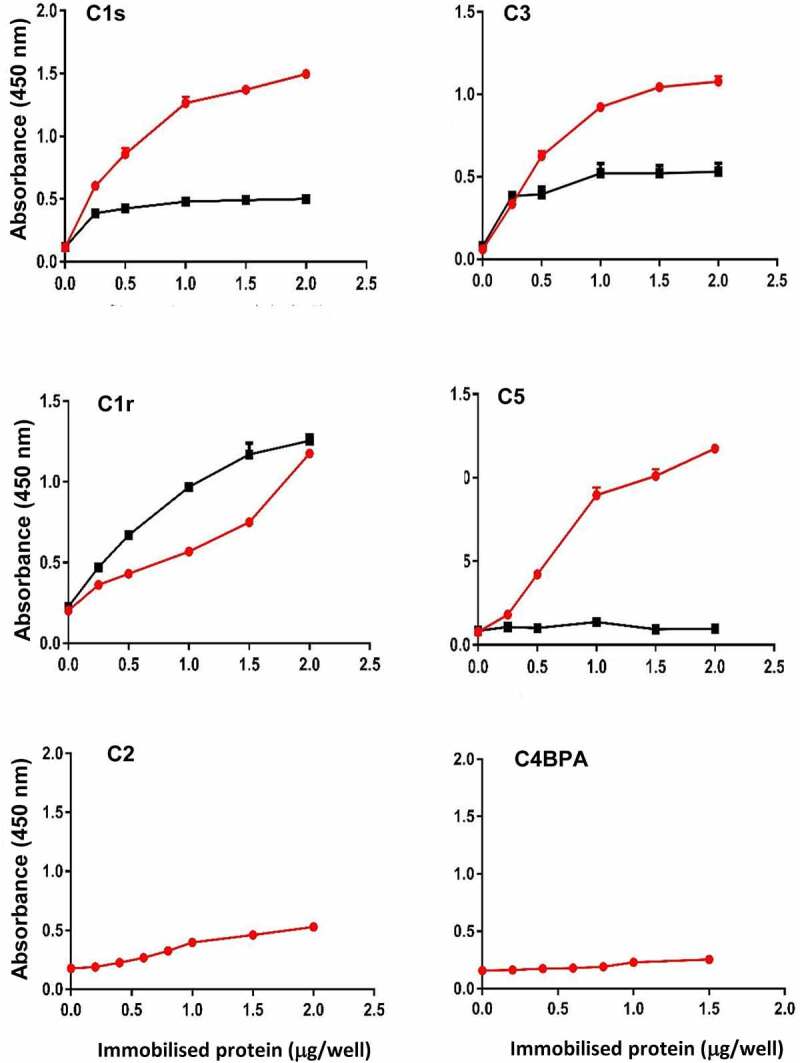
**CEF binds to complement proteins**. Binding of complement proteins C1r, C1s, C2, C3, C5 and C4BPA was analyzed by ELISA using immobilized rCEF. Proteins were treated or untreated with PNGase F to remove N-linked glycans. Dose-dependent binding was detected using specific primary antibodies and a secondary HRP-conjugated anti-rabbit IgG antibody. Red line: before PNGase F treatment. Black line: after PNGase F treatment. Data represent the mean ± SD of three independent experiments in duplicates.

To confirm the specific binding, rCEF was immobilized on ELISA plates, probed with commercially obtained complement proteins C1r, C1s, C3, and C4BPA and developed with complement protein-specific antibodies. We also analyzed the binding to complement proteins C2 and C5, which were not found in the pull-down assay. All complement proteins, except C2 and C4BPA, showed dose-dependent binding to rCEF (Figure 2). Based on the range of binding partners, the lack of amino acid homology between the complement proteins, and the fact that most complement factors are glycoproteins [43], we hypothesized that CEF might bind to a conserved glycan. Indeed, after treatment with PNGase F, an amidase that removes N-linked sugars from glycoproteins [44], binding to rCEF was substantially reduced for C1s and C3 and almost completely abolished for C5. However, PNGase F treatment failed to reduce binding of rCEF to C1r, but instead slightly increased binding (Figure 2).

### CEF interferes with complement activity

**Figure 3.**
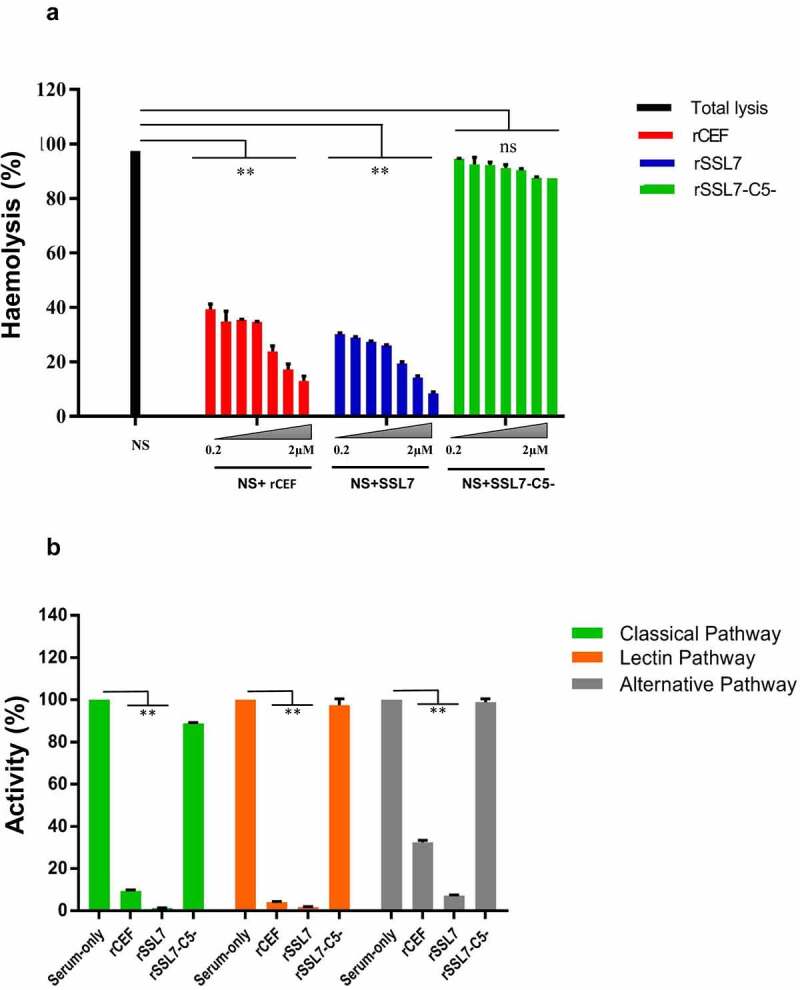
**rCEF interferes with complement function**. A. Complement-mediated lysis of red blood cells (RBCs). rCEF significantly reduced hemolysis of RBCs in a dose-dependent manner. *Staphylococcus aureus* SSL7, a known complement inhibitor [45] was used as a positive control. The nonfunctional mutant SSL7-C5^−^[46] was used as a negative control. Data represent hemolysis as mean ± SD of triplicate A_412nm_ readings. **p < 0.0001, unpaired two-tailed t-test. B. Wieslab human complement activity assay. Activation of individual complement pathways was assessed using the Wieslab® complement system screen kit. rCEF, SSL7 (positive control), and SSL7-C5^−^ (negative control) were tested at a concentration of 2 µM. The interference with the activity of each of the three complement pathways is shown in comparison to the control serum without the added proteins. The C5b-9 complex (membrane attack complex, MAC) was detected with a specific alkaline phosphatase labeled antibody to a neoantigen of the MAC complex. Analyzing the result by one-way ANOVA revealed significant differences (**p < 0.0001) between the marked groups. No significant difference was observed between the negative control and serum-only group. Data are presented as means ± SD for two independent experiments performed in duplicate.

To test if CEF binding to complement proteins affected complement activity we used a standard hemolysis assay. Addition of 0.2 μM rCEF reduced hemolysis by about 60%, and only about 10% activity was observed after addition of 2 μM rCEF (p < 0.0001, Figure 3a). These results were comparable with the activity of staphylococcal superantigen-like protein 7 (SSL7) from *Staphylococcus aureus*, a known inhibitor of complement function [45]. In contrast, the nonfunctional SSL7-C5- mutant [46] only marginally reduced hemolysis to just under 90% at a concentration of 2 μM.

Next, we investigated which of the three complement pathways were affected by CEF using the commercial Wieslab assay kit. The addition of 2 µM rCEF resulted in a significant reduction of activity in all three pathways (p < 0.0001). The CP and LP activity saw the greatest reduction (<10% activity), whereas the AP was reduced to around 30% activity (Figure 3b). This was similar to the effect seen after the addition of 2 µM SSL7, which also reduced the activity of the CP and LP (<5%) and to a lesser extent, the AP (<10%). The SSL7 C5-mutant was added at 2 µM as negative control and failed to inhibit the LP and AP but slightly inhibited the CP (approximately 90% activity).

**Figure 4.**
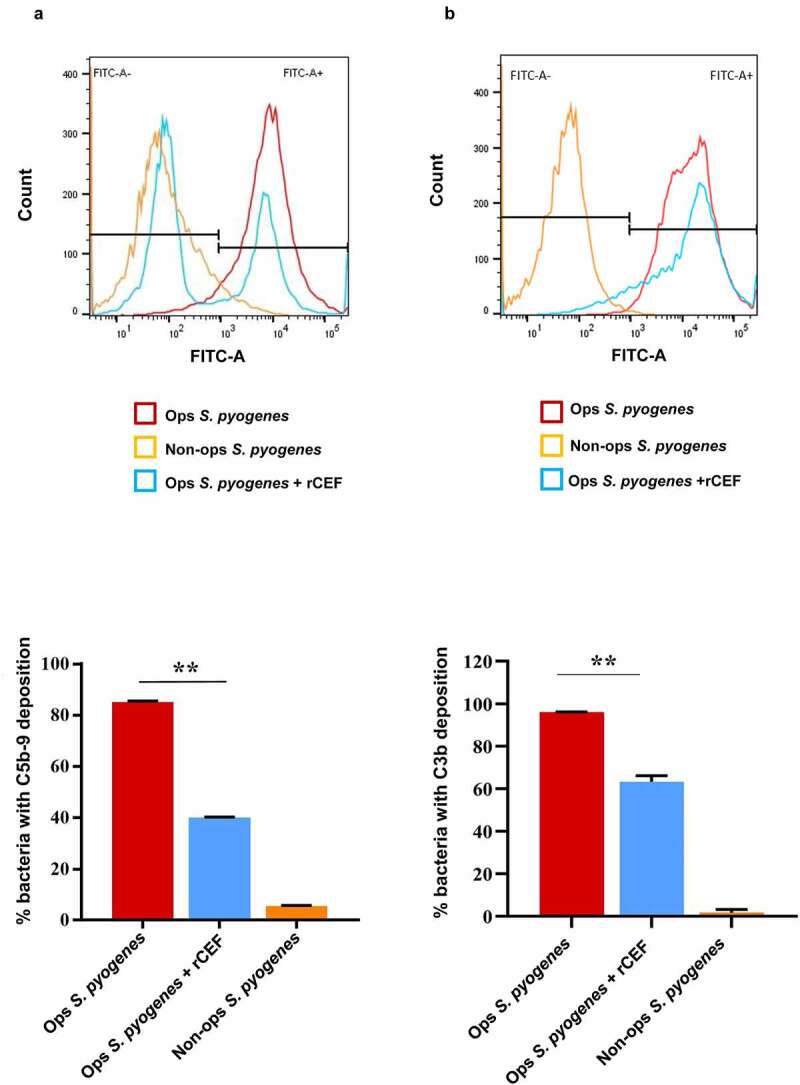
**CEF inhibits deposition of MAC and C3b on the surface of *S. pyogenes***. The deposition of MAC (C5b-9) (a, b) or C3b (c, d) on the surface of *S. pyogenes* was analyzed by flow cytometry. The bacteria were opsonised with 10% human serum in the absence (Ops *S. pyogenes*) or presence of 0.5 μM rCEF (Ops *S. pyogenes* + rCEF). Non-opsonised (Non-ops *S. pyogenes*) were used as a control. (a, c) The flow cytometry histogram shows an overlay of bacteria incubated with human serum (red), buffer (Orange), or serum + rCEF (blue). A gate has been applied to identify the percentage of C5b-9 or C3b deposition (FITC-A^+^). Bar graph displaying the percentage of bacterial cells positive for C5b-9 (b) or C3b (d). Analyzing the result by one-way ANOVA revealed significant differences between the groups (**p < 0.0001).

To analyze, if CEF also interferes with MAC formation on the surface of a bacterium, we carried out a complement deposition assay using *S. pyogenes* and human serum as a source of complement. MAC formation was analyzed by flow cytometry using a specific anti-C5b-9 antibody. Addition of 0.5 µM rCEF resulted in a > 50% decrease in MAC formation on the bacterial cell surface (p < 0.0001, Figure 4a). The functional role of MAC formation on the surface of Gram-positive bacteria is unclear as it was shown that cells are not lysed due to the thick peptidoglycan layer which prevents contact of MAC with the bacterial cell membrane [47]. We therefore also tested if CEF is able to prevent opsonization with C3b, a crucial step for phagocytosis. We found that rCEF also inhibited C3b deposition, although to a lesser extent than MAC deposition (approximately 30% decrease, p < 0.0001) (Figure 4b).

### CEF plays a crucial role in S. pyogenes virulence

**Figure 5.**
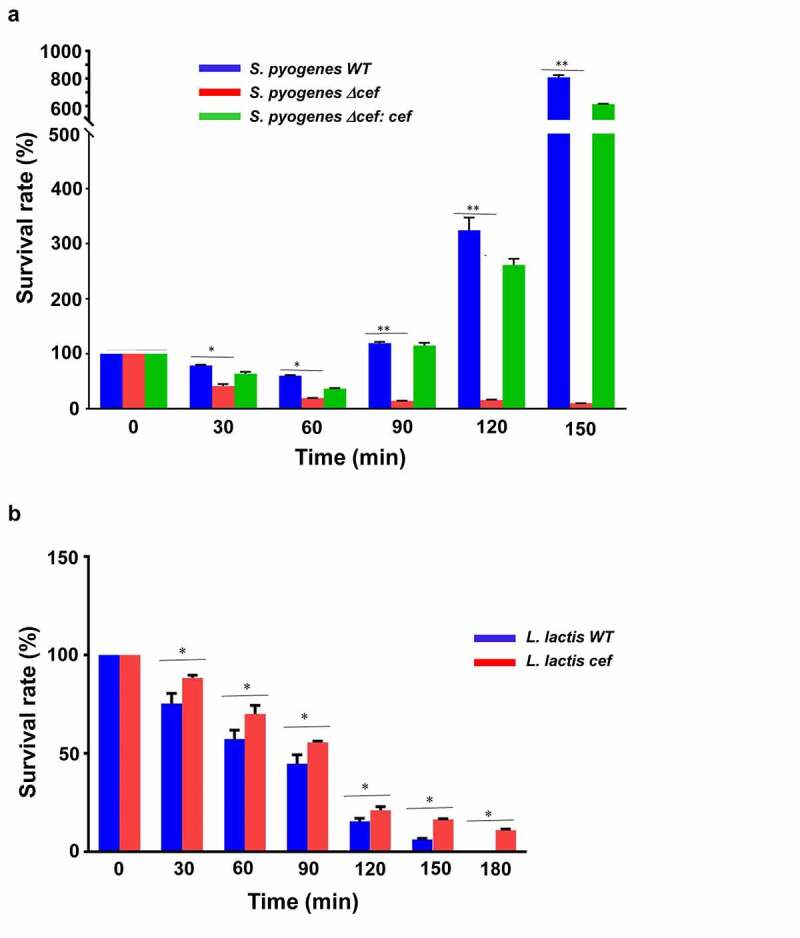
**CEF promotes bacterial survival in human blood**. (a) Survival rate of *S. pyogenes* wild-type (WT), *S. pyogenes* Δ*cef* deletion mutant, and *S. pyogenes* Δ*cef:cef* complementation strain after incubation with whole human blood. Deletion of *cef* caused a considerably reduced survival rate compared to the CEF expressing strains. (b) Survival of *L. lactis* wild-type (WT) and a *L. lactis cef* gain-of-function mutant in whole human blood. The mutant strain showed an improved survival rate compared to wild-type *L. lactis*. Survival rates were calculated by dividing the CFU at a given time point by the CFU of the initial inoculum. The data shown are representative of three independent experiments, displayed as the mean ± SD. Significant differences between strains were calculated by Student’s *t*-test, *p < 0.05, **p < 0.0001.

To demonstrate that CEF plays a crucial role in *S. pyogenes* virulence we first generated a *S. pyogenes cef* deletion mutant by allelic replacement with the spectinomycin resistance gene *aad9*, followed by complementation with the wt *cef* gene. Expression of the *cef* gene was analyzed by Western blot analysis with cell culture supernants from *S. pyogenes* and the mutated strains grown to late exponential phase (OD_600nm_ ~ 0.8). The *S. pyogenes Δcef* mutant lacked any expression of CEF, which was partially restored in the *S. pyogenes Δcef:cef* strain (Supplementary Figure S1). In addition, we generated a gain-of-function mutant using the non-virulent food-grade bacterium *Lactococcus lactis*. The WT and mutant strains were analyzed in a whole blood killing assay. Survival of the *S. pyogenes Δcef* mutant decreased by >90% from a starting amount of ~10^5^ CFU to under 10^4^ CFU after 150 min. In contrast, *S. pyogenes* WT showed an approximately 8-fold increase in growth during the same time and the *S. pyogenes Δcef:cef* mutant partially complemented the phenotype showing an approximately 6-fold increase in growth after 150 min (Figure 5a). In contrast, *L. lactis* WT didn’t survive in human blood and was completely killed after 180 min. Introduction of *cef* in the gain-of-function *L. lactis cef* mutant delayed killing, with 11% survival still remaining after 180 min suggesting a role of CEF in virulence (Figure 5b).

**Figure 6.**
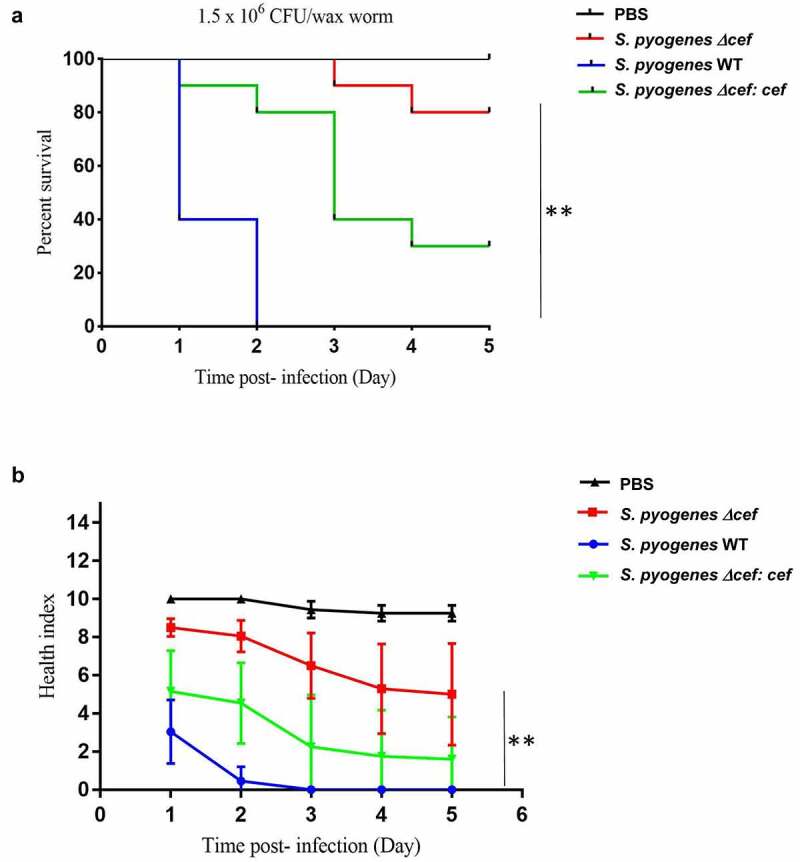
**CEF is a crucial virulence factor in a *Galleria mellonella* wax moth larvae infection model**. Antibiotic-free *G. mellonella* larvae (n = 10) were injected with 20 µl (1.5x10^6^ CFU) of *S. pyogenes* WT, *S. pyogenes Δcef* or *S. pyogenes Δcef:cef* into the lower-left proleg and monitored daily for 5 days. A. Survival of larvae is shown as Kaplan-Meier survival curves. **p < 0.0001 (log-rank test) B. Health index scores for infected larvae. Data shows the mean ± SEM, **p < 0.0001 (2-way ANOVA).

The role of CEF in *S. pyogenes* virulence was further tested in a *Galleria mellonella* infection model. This insect model has been introduced as an alternative model to study microbial infections. Although insects lack an adaptive immune response, their innate immune response shows remarkable similarities with the immune response in vertebrates, including the presence of complement proteins [48]. We have recently shown that *G. mellonella* is a useful model to investigate *S. pyogenes* virulence [49]. *G. mellonella* larvae (n = 10 per group) were infected with 1.5 × 10^6^ CFU of *S. pyogenes* WT, *S. pyogenes Δcef* or *S. pyogenes Δcef: cef* and monitored over a period of five days (Figure 6). Survival of larvae dropped to 0% only 2 days after infection with *S. pyogenes* WT. In comparison, the survival rate of larvae infected with *S. pyogenes Δcef* remained high throughout the experiment (80% on day 5, p < 0.0001). Complementation partially restored virulence of *S. pyogenes Δcef*, with 30% survival remaining on day 5 of larvae infected with *S. pyogenes Δcef:cef* (Figure 6a). Health index scoring of *G. mellonella* which monitors disease signs such as larvae mobility, cocoon formation and melanization in addition to death [48] were consistent with survival results. Larvae infected with *S. pyogenes Δcef* achieved substantially higher health scores compared to larvae infected with *S. pyogenes* WT (p < 0.0001), whereas larvae infected with *S. pyogenes Δcef: cef* had intermediate scores indicating that the complementation with *cef* could partially restore the WT phenotype (Figure 6b). These results provide further evidence that CEF plays a crucial role as *S. pyogenes* virulence factor.

### CEF is produced by S. pyogenes during disease

**Figure 7.**
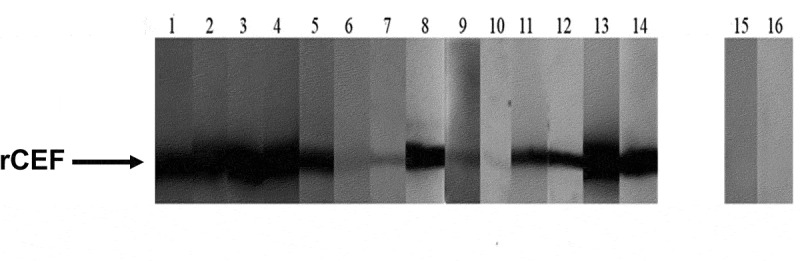
**Specific anti-CEF antibodies in serum from patients with *S. pyogenes* disease**. Recombinant CEF (2 μg/lane) was run on a 12% SDS-PAGE gel and blotted onto a nitrocellulose membrane. Individual strips of the Western blot were probed with serum from *S. pyogenes* invasive disease patients (lanes 1–14) or healthy donors (lanes 15–16) and developed with a secondary HRP-conjugated anti-human Fc antibody.

The presence of specific antibodies against a protein produced by a pathogen is a useful indicator of protein expression during infection. Purified rCEF was used to probe a panel of invasive disease patient sera (n = 14) and healthy control sera (n = 2) using Western blot analysis. Ten of the 14 disease sera showed moderate-to-strong activity against rCEF, whereas 3 sera showed only very weak activity (Figure 7). None of the healthy donor sera showed any activity against rCEF. This strongly suggests that CEF is produced during invasive GAS disease.

## Discussion

With the aim to identify novel *S. pyogenes* virulence factors, we searched the *S. pyogenes* genome downstream of the variable FCT-region which encodes several virulence proteins, including fibronectin-binding proteins and all the elements required to assemble the *S. pyogenes* pilus [40–42]. We identified an open reading frame (*spy0136*) encoding for a hypothetical protein of unknown function that was predicted to be secreted due to the presence of an N-terminal signal peptide sequence. A pull-down experiment with immobilized recombinant Spy0136 and human plasma, combined with mass-spectroscopy, resulted in the identification of several Spy0136 binding partners, including fibrinogen and complement proteins C1r, C1s, C3 and C4BPA. Hence, Spy0136 was named “complement evasion factor” (CEF). Binding to these plasma proteins was confirmed by ELISA with the exception of C4BPA which failed to bind to CEF in the ELISA. Binding to C5 was also detected by ELISA, but not by the pull-down assay. Given the promiscuous binding to a variety of proteins, we speculated that CEF binding might be mediated through conserved glycans. Indeed, removal of N-linked glycans with PNGase F abrogated CEF binding to C1s, C3 and C5, but not to C1r. Binding of CEF to C1r may be glycan-independent or involve binding to O-linked glycans which weren’t removed by PNGase F. Glycan-dependent binding might also explain the reason why C4BPA failed to bind rCEF in the ELISA despite the observed binding in the pull-down experiment. In contrast to C1r, C1s, C3 and C5, which were purchased as proteins purified from human plasma, C4BPA is not commercially available as a native protein and was manufactured from an *in-vitro* wheat germ expression system that is unable to glycosylate proteins. On the other hand, C5 was not identified in the pull-down assay but bound to rCEF in the ELISA. It is possible that C5 was enriched from the human serum sample but was one of the protein bands that failed to give mass-spectroscopy results. Alternatively, C5 might compete for CEF-binding with C3 which is far more abundant in human plasma. Notably, treatment of C1r with PNGase F increased binding to CEF, although only slightly. A possible explanation might be that the removal of N-linked glycans provided better access to the actual CEF binding site which might be partially masked by the N-linked carbohydrate.

In order to demonstrate that CEF binding to the identified targets was functional, we analyzed rCEF interference with complement-mediated lysis of erythrocytes. rCEF was able to prevent complement-mediated erythrocyte lysis similar to the *S. aureus* complement inhibitor SSL7, which also binds C5 to prevent MAC formation and neutrophil chemotaxis, possibly by inhibiting C5 cleavage to C5a (a chemoattractant) and C5b (a prerequisite for MAC formation). To further decipher which complement pathway is affected by CEF, we used a commercial Wieslab kit, which allows each of the complement pathways to be triggered independently. rCEF interfered with all three pathways, although to a lesser extent with the alternative pathway. The classical pathway is triggered by Ig opsonising the pathogen surface resulting in deposition of C1q, C1r, and C1s (C1 complex). C1r activates C1s, which in turn splits C2 and C4 to form the CP C3 convertase (C4bC2b complex). Binding of CEF to both C1r and C1s might therefore explain the strong interference with the classical pathway. Binding of CEF to C3 might interfere with the C3 convertase-controlled split into C3a and C3b, followed by C3b opsonization of the pathogen for phagocytotic killing. This is in line with the result of our C3b deposition assay that showed approximately 30% inhibition by rCEF. However, it is not entirely clear why the alternative pathway is affected less than the other two complement pathways as C3 cleavage is common to all three pathways. A possible explanation might be that C3 binding and cleavage by the alternative pathway (or fluid phase) C3 convertase is less affected by CEF than the other C3 convertases, but this requires future investigation. Like SSL7, CEF also binds to C5 which is cleaved by C5 convertase to form C5a and C5b, before C5b binds to additional complement proteins to form the MAC. The alternative pathway C5 convertase is again different from the classical pathway C5 convertase which might explain the differences in CEF interference with the alternative pathway. This was also observed, although to a lesser extent, with SSL7 but not with a mutant form (SSL7-C5-) that is defective in binding to C5 but not IgA [46]. Inhibition of MAC formation was shown for all 3 complement pathways in the Wieslab assay and we also showed CEF-mediated inhibition of MAC formation on the bacterial surface. Interference with MAC formation is also a function of *S. pyogenes* streptococcal inhibitor of complement (SIC) [30] and *S. aureus* SSL7 [50]. Why Gram-positive bacteria would target MAC formation is perplexing, as it is believed that the thick peptidoglycan layer in the cell wall would prevent MAC from reaching the membrane and form lytic pores. However, it has been shown that in contrast to C3b deposition which occurs randomly on the bacterial surface, C5b-9 (MAC) deposition occurs at specialized regions on *S. pyogenes*, depositing near the division septum. This suggests that the bacteria might be sensitive to the insertion of C5b-9 during specific growth phases due to the dynamic structure of the cell wall [51].

It is also possible that inhibition of MAC formation might simply be the consequence of CEF interference(s) earlier in the complement cascade. For example, if binding of CEF to C3 prevents the generation of C3b, this would prevent the formation of the C5 convertases (e.g. C4b2a3b) and consequently the formation of C5b, which is necessary for MAC formation. CEF might also have additional yet undiscovered binding partners apart from complement proteins similar to SIC which also inhibits the antibacterial activity of a wide range of antimicrobial peptides and proteins, including lysozyme, LL-37 and human beta-defensins [52]. SIC also interacts with both human thrombin and plasminogen to inhibit fibrinolysis [53] and with Toll-like receptor 2 (TLR-2) and CD14 on monocytes to induce pro-inflammatory cytokines [54].

The variety of immune evasion functions of already-known virulence proteins posed the question, is CEF crucial for *S. pyogenes* virulence and not simply a redundant mechanism? This was addressed by testing an isogenic *cef* deletion mutant (*S. pyogenes Δcef*) and its complemented strain (*S. pyogenes Δcef:cef*). We also tested a gain of function *cef* mutant in the nonpathogenic *L. lactis* (*L. lactis cef*), a bacterium that normally lacks any virulence factors. A whole blood killing assay and a *G. mellonella* larvae infection model showed that the *cef* deletion mutant was less virulent compared to the *S. pyogenes* WT strain. The virulence phenotype could be restored when the *cef* mutant was complemented with the *cef* gene, but only partially. The partial complementation might be due to CEF expression levels as the *cef* gene was put under the control of a constitutive *S. pyogenes* promotor which is part of the pLZ12km expression plasmid. A future study will aim at the identification of the native *cef* gene promotor which will then be used to drive CEF expression in the complement strain. The significant phenotypic difference between the *L. lactis* gain-of-function mutant and *L. lactis* WT strain in the whole blood killing assay provides further evidence that CEF plays an important role in *S. pyogenes* virulence.

A possible reason for the production of various virulence factors with overlapping functions might be an adaptation to certain stages of disease. It has previously been shown that the human antimicrobial peptide LL-37 triggers upregulation of *spy0136/cef* in addition to well-known virulence genes, such as *speA* (streptococcal pyrogenic exotoxin A), *sda1* (streptococcal dornase 1), *ska* (streptokinase), *slo* (streptolysin O), and *nga* (NAD-glycohydrolase), which are all under the control of CsrRS (or CovRS), a two-component system that controls expression of up to 15% of the *S. pyogenes* genome [55]. It was hypothesized that CsrRS mediates conversion of *S. pyogenes* from a colonizing to an invasive phenotype in response to signaling by host LL-37^55^. This is also in line with the results of a transcriptomic study in a mouse soft tissue infection model which demonstrated that *spy0136/cef* was expressed during infection and upregulated >13-fold in a *covRS* mutant strain [56]. Further evidence was provided by Aziz and colleagues who looked at differences in the secreted proteomes of SpeB expressing strains versus SpeB- strains and observed that expression of active SpeB caused the degradation of the majority of secreted *S. pyogenes* proteins, including several known virulence factors. They proposed that the invasive *S. pyogenes* strain undergoes an *in-vivo* phase shift to prevent proteolytic degradation of multiple virulence factors by SpeB [57]. This hypervirulent *speB-* strain arises *in-vivo* due to the selection of bacteria with mutations in *covS*, the sensor part of the CovR/S system [58]. This would suggest that *cef* is also upregulated during invasive disease in the absence of SpeB and is further supported by evidence from a transcriptome study in *S. pyogenes* infected cynomolgus macaques which showed a strong correlation of *spy0136/cef* expression with peak levels of C-reactive protein, a sensitive serum marker of inflammation, and a negative correlation with the development of pharyngitis and tonsillitis [59]. These findings are in line with our observation that invasive disease patients produced antibodies against CEF. Future studies with a larger number of serum samples, including from patients with noninvasive diseases such as pharyngitis might shed further light on the role of CEF in *S. pyogenes* disease.

In conclusion, we have identified a novel complement evasion factor (CEF) that interacts with at least four different complement proteins to inhibit all three pathways of the complement system. This is the first *S. pyogenes* virulence factor with promiscuous binding to glycoproteins via glycan interactions. The high conservation and wide strain distribution of the *cef* gene and immunogenicity of the secreted CEF protein indicates a potential target for vaccine development. However, further studies need to address the functionality of the antibodies to determine if they provide protection against *S. pyogenes* disease.

## Supplementary Material

Supplemental Material

## Data Availability

The authors confirm that the data supporting the findings of this study are available within the article. Additional data are available from the corresponding author [TP] upon reasonable request.
